# Contributions to Mental Pathology

**Published:** 1873-04

**Authors:** 


*Contributions to Mental Pathology.* By I. RAY, M. D. Boston: Little, Brown & Co., 1873. Buffalo: James M. Lent.

With but two exceptions the papers comprising this book have appeared in print. But as the points of which some of them treat, are still unsettled, and as all are of interest to those interested in insanity, the author has placed them before the public in book form. Without claiming to be a treatise on Insanity in any of its forms, it is a contribution to the subject which can not but be received with pleasure by the profession. Several articles are upon important trials where insanity was claimed by one party or the other; the author's own opinion is given in some of the cases, and affords evidence of a profound knowledge of the subject.

Perhaps to the non-professional reader, the articles on the insanity of King George, Shakespeare's illustration of Insanity, and illustrations of insanity by distinguished English writers, will be the most interesting.

We have been greatly interested and instructed in their perusal, and consider his review of the writings of Shakespeare as unexampled for a profound knowledge of the subject and a perfect acquaintance of the author's ideas.

The book comprises over five hundred and fifty pages, and constitutes a valuable contribution to the literature of Insanity.

